# Satellite assessment of winter cover crop and conservation tillage outcomes to support adaptive management in working landscapes

**DOI:** 10.1002/jeq2.70082

**Published:** 2025-10-21

**Authors:** W. Dean Hively, Feng Gao, Gregory W. McCarty, Craig S. T. Daughtry, Xuesong Zhang, Jyoti Jennewein, Alison Thieme, Brian T. Lamb, Jason Keppler, Cathleen J. Hapeman, Michael Cosh, Steven B. Mirsky

**Affiliations:** ^1^ U.S. Geological Survey Lower Mississippi‐Gulf Water Science Center Beltsville Maryland USA; ^2^ USDA‐ARS Hydrology and Remote Sensing Laboratory Beltsville Maryland USA; ^3^ USDA‐ARS Sustainable Agricultural Systems Laboratory Beltsville Maryland USA; ^4^ Maryland Department of Agriculture Office of Resource Conservation Annapolis Maryland USA

## Abstract

The use of winter cover crops and conservation tillage are agricultural practices promoted to reduce nutrient and sediment loss from cropland, improve soil health, increase infiltration, and support farm nutrient cycling and ecosystem services. However, environmental performance of these practices is variable in the working farm landscape. The Lower Chesapeake Bay research project within the USDA Long‐Term Agroecosystem Research (LTAR) network has collaboratively developed satellite remote sensing algorithms to measure the performance and phenology of winter cover crops (aboveground biomass, nitrogen content, fractional cover, and emergence and termination dates) using no‐cost Harmonized Landsat and Sentinel‐2 multispectral satellite imagery. This research supports annual operational assessment of >28,000 fields per year in four states. Results document the impacts of agronomic management on conservation outcomes, support adaptive management of incentive payment structures, and can reduce the workload for conservation district staff by remotely verifying cover crop management. Additionally, super‐spectral satellite applications have been developed to accurately map crop residue cover by measuring lignocellulose absorption in shortwave infrared wavelengths, producing a 7‐year time series of tillage intensity maps for the Delmarva Peninsula. These remote sensing products can be used in decision support and modeling to estimate changes in nutrient, sediment, and carbon cycling resulting from conservation practice implementation in the working farm landscape. This manuscript provides an overview of remote sensing research findings and applications associated with the USDA LTAR and Conservation Effects Assessment Projects (CEAP), documenting a variety of previously published outcomes with update and expansion of techniques using additional unpublished data and analyses as appropriate.

## Core Ideas

- The United States Department of Agriculture Long Term Agroecosystem Research network and Conservation Effects Assessment projects have developed remote sensing tools to support agricultural conservation implementation.
- Satellite‐based measurement of cover crop performance informs adaptive management of incentive programs.
- Annual operational remote sensing analysis assists Maryland Department of Agriculture cover crop program management.
- Crop residue cover is accurately estimated by measuring lignocellulose absorption features in the shortwave infrared wavelengths.
- Rapidly advancing technology will soon provide global‐scale super‐spectral and hyperspectral satellite imagery.

## Plain Language Summary

The Lower Chesapeake Bay Long Term Agricultural Research project, in collaboration with the USDA Conservation Effects Assessment Project and the US Geological Survey (USGS) National Land Imaging program, has developed methods to measure performance of agricultural conservation practices at field and landscape scales. These tools were developed through >10 years of research and use satellite imagery to measure and map winter cover crop biomass, nitrogen content, and fractional ground cover throughout the winter growing season, and to estimate dates of cover crop green‐up and termination. This information is used operationally by the Maryland Department of Agriculture to inform adaptive management of cover crop incentive programs. Additionally, narrow‐band SWIR satellite imagery obtained through USGS is used to measure fractional crop residue cover and to map the impact of conservation tillage at the landscape scale. Applied remote sensing conducted in partnership with conservation program managers can support adaptive management of conservation programs to increase environmental impact.

**Abbreviations**
ARS: Agricultural Research Service
BARC: Beltsville Agricultural Research Center
CAI: cellulose absorption index
CEAP: USDA Conservation Effects Assessment Project
EMIT: Earth Surface Mineral Dust Source Investigation imaging spectrometer aboard the International Space Station
fCRC: fraction of crop residue cover on the soil surface
HLS: Harmonized Landsat Sentinel‐2
LCB: Lower Chesapeake Bay
LTAR: USDA Long Term Agroecosystem Research network
NASA: US National Aeronautics and Space Administration
NDVI: normalized difference vegetation index
NPV: non‐photosynthetic vegetation
PRISMA: *PRecursore IperSpettrale della Missione Applicativa* spaceborne imaging spectrometer
S2NG: Sentinel‐2 Next Generation
SWIR: shortwave infrared
USDA: United States Department of Agriculture
USGS: US Geological Survey
WCC: winter cover crop

## 1 INTRODUCTION

### 1.1 Long term agroecosystem research

The USDA Long Term Agroecosystem Research network (LTAR) is led by the Agricultural Research Service (ARS) of United States Department of Agriculture (USDA) and consists of 19 research sites across the United States that have several common research goals (Tsegaye et al., 2024), many of which follow on the USDA Conservation Effects Assessment Project (CEAP) Watershed Assessment Studies. The sites share the mission “to conduct long‐term, transdisciplinary, networked research to create innovative tools and practices, with regionally tailored evidence‐based knowledge supporting adaptable, resilient, sustainable agriculture.” The largest network activity engages 14 of the research sites in a Common Experiment that compares regionally specific prevailing production systems to alternative or aspirational management practices using a set of common indicators (Liebig et al., 2024). The goal of the alternative/aspirational production methods is to improve the sustainability of the overall farming system and to develop new technologies and markets in concert with changing societal expectations. Most of the 14 cropland Common Experiments include the use of cover crops and conservation tillage in their alternative/aspirational production practices with the goal of achieving increased carbon sequestration, decreased loss of nutrients and sediment, and overall improved water quality. That includes the Common Experiment site managed by the Lower Chesapeake Bay (LCB) LTAR, located at the USDA‐ARS Beltsville Agricultural Research Center (BARC), Beltsville, MD, as presented in Bagley et al. (2024). Ultimately, the LTAR Network seeks to ensure the relevancy and adoption of aspirational research efforts by working directly with farmers and with state agricultural and environmental departments throughout the research process.

### 1.2 The Chesapeake Bay watershed and the lower Chesapeake Bay LTAR site

The Chesapeake Bay watershed is the largest estuary in the United States (16.6 million ha), supports over 18.5 million people and nearly 36,000 species of flora and fauna, and has the world's largest land‐to‐water ratio of any enclosed waterbody (14:1). The Chesapeake Bay receives tidal waters from the Atlantic Ocean and fresh water from twelve major tributaries (Chesapeake Bay Program, n.d.a, n.d.b). Decades of pollution from many sources led to the formation of the Chesapeake Bay Program in 1983, and the signing of a Chesapeake Bay Watershed Agreement in 2014 establishing state‐level water quality restoration objectives. Included in these goals are reductions in nutrient and sediment runoff from agriculture to be achieved through implementation of a suite of agricultural best management practices including cover cropping, conservation tillage, nutrient management planning, manure and poultry litter management, and riparian buffers (Chesapeake Bay Program, n.d.d).

The LCB LTAR project (https://ltar.ars.usda.gov/sites/lcb/) conducts field‐ and landscape‐scale research in Maryland, Delaware, and Pennsylvania, with a primary focus in Beltsville, MD, and on farmland on the Delmarva Peninsula (Maryland and Delaware) within the Atlantic Coastal Plain environment. The LCB research area also includes land directly adjacent to the estuarine areas of the Chesapeake Bay watershed, below the fall line of the major tributaries, and also includes the adjacent Delaware Bay watershed due to similarities in soil, drainage, and land use (Bean et al., 2021). The LCB LTAR project draws on the ongoing research efforts of the USDA Choptank River Watershed CEAP, initiated in 2004 in partnership with USDA Natural Resources Conservation Service, and includes collaboration with the US Geological Survey—National Land Imaging and Land Change Science programs. A major research thrust of the Choptank River Watershed CEAP (https://www.ars.usda.gov/anrds/ceap/maryland‐choptank/) has been to examine the implementation and performance of winter cover crops in cooperation with the Maryland Department of Agriculture (MDA), as well as to map the impacts of crop residue cover and conservation tillage adoption.

### 1.3 The Maryland Department of Agriculture winter cover crop program

Each state in the Chesapeake Bay watershed works to meet their water quality objectives in part by encouraging implementation of agricultural conservation practices. Cover crops are planted to provide living cover in the winter months between summer cropping seasons and have multiple benefits for soil health and ecosystem services (Wood & Bowman, 2021). In the Chesapeake Bay region, winter cereal cover crop species such as rye (*Secale cereale* L.), barley (*Hordeum vulgare* L.), triticale (*Triticale hexaploide* Lart.), and wheat (*Triticum aestivum* L.) are planted primarily to prevent nitrogen leaching in the hydrologically active winter period (Gray & Heide, 2013). The MDA created the Maryland Agricultural Cost Share (MACS) program in 1997 to incentivize farmers to adopt best management practices including cover crops, to reduce nutrient and sediment loss from agriculture (Chesapeake Executive Council, 2014; Meisinger et al., 1991). Maryland, along with its neighboring states in the Chesapeake Bay watershed, now leads the nation in cover crop implementation (41% of commodity cropland area in 2017; Myers & LaRose, 2022). MACS cost‐share payments include a base payment per field area, with additional incentive payments associated with planting dates, planting methods, species, and previous crop. This program supports farmers in planting >160,000 ha (>400,000 acres) of cover crops every fall (Maryland Department of Agriculture, 2018). Each year, the MDA evaluates the incentive structure, implementation trends, and current cover crop research and adjusts incentive rates to promote management practices associated with higher environmental benefits (Thieme et al., 2023).

**Core Ideas**

- The United States Department of Agriculture Long Term Agroecosystem Research network and Conservation Effects Assessment projects have developed remote sensing tools to support agricultural conservation implementation.
- Satellite‐based measurement of cover crop performance informs adaptive management of incentive programs.
- Annual operational remote sensing analysis assists Maryland Department of Agriculture cover crop program management.
- Crop residue cover is accurately estimated by measuring lignocellulose absorption features in the shortwave infrared wavelengths.
- Rapidly advancing technology will soon provide global‐scale super‐spectral and hyperspectral satellite imagery.

### 1.4 Background on cover crop remote sensing research

Plot‐scale research has documented substantial reductions in nutrient and sediment loss associated with planting cover crops (Dabney et al., 2010; Staver & Brinsfield, 1998). However, these studies do not capture the variety of environmental outcomes that result from variable cover crop management on working farms. Satellite remote sensing, combined with site‐specific agronomic management information for fields enrolled in incentive programs, can support the analysis of cover crop performance at landscape scale (Hively et al., 2009; Hively, Duiker, et al., 2015).

One reliable source of multispectral satellite imagery, available with regularity at the regional scale, is the Harmonized Landsat Sentinel‐2 (HLS) dataset (Claverie et al., 2018). This freely available product integrates well‐calibrated, cloud‐masked imagery from US National Aeronautics and Space Administration (NASA) and the European Space Agency (ESA) to provide global coverage at 30‐m pixel resolution every 3–5 days. Spectral indices such as the normalized difference vegetation index (NDVI; Rouse et al., 1973; Tucker, 1979) and the deltaRE red edge index (Reusch, 2005) are strongly correlated with fractional green vegetative cover, aboveground biomass, and nitrogen uptake of winter cover crops (Jennewein, et al., 2022; Thieme et al., 2023, 2020, 2025).

The MACS program maintains a privacy‐protected geospatial database of fields annually enrolled in its winter cover crop program, along with associated field management data, that our research team is able to access through a cooperative research agreement among MDA, U.S. Geological Survey, and USDA‐ARS. By combining the field location and management information with the satellite imagery for each field, in a privacy‐protected context, cover crop performance can be linked to field management and incentive payments and provide map‐based analyses to inform conservation decision making (Hively et al., 2024). These methods are described in greater detail in this manuscript.

### 1.5 Background on crop residue remote sensing research

In addition to winter cover crops, Chesapeake Bay state agencies promote conservation tillage to reduce nutrient and sediment loss from farmland (USEPA, 2024). Tillage systems can be characterized by the fraction of crop residue cover on the soil surface (fCRC) in the springtime immediately following planting of the summer crop. Crop residue includes various types of non‐photosynthetic vegetation (NPV), most often debris left on the field following crop harvest, such as stalks and debris from corn (*Zea Mays* L.), soybean (*Glycine max* L.), and wheat, but also including senesced biomass of cover crops and weeds. The residue provides a mulch on the soil surface that reduces raindrop impact and sediment entrainment while also increasing infiltration, moderating soil temperature and evaporation, and benefitting soil health (Palm et al., 2014). The Chesapeake Bay Program Partnership defines four categories of tillage intensity: plow till (0%–15% fCRC), reduced tillage (15%–30% fCRC), conservation tillage (30%–60% fCRC), and high residue no‐till systems (60%–100% fCRC) (Chesapeake Bay Program, n.d.e).

Crop residue cover is traditionally measured in the field in the springtime, using line‐point transects or photo‐based methods, which can be costly and time‐consuming (Hively et al., 2018). Increasingly, satellite imagery is being used to estimate fCRC using multispectral indices such as the normalized tillage difference index (NDTI; van Deventer et al., 1997) and shortwave infrared angle index (SWIRA; Yue et al., 2022) that can be derived from Landsat and Sentinel‐2. However, these measurements are most accurate under dry conditions and degrade rapidly with increasing moisture content (resulting from precipitation) and increasing presence of green vegetation (which masks the field surface and has a reflectance pattern similar to crop residue when measured using the broadband shortwave infrared [SWIR] reflectance data available from Landsat [180 nm bandwidth] and Sentinel‐2 [94 nm bandwidth]).

Crop residue cover is more accurately characterized by measuring lignocellulose absorption features in the SWIR using narrow‐band indices such as the cellulose absorption index (CAI; Daughtry et al., 2006; Nagler et al., 2000) and the shortwave infrared normalized difference index (SINDRI; Daughtry et al., 2018; Serbin et al., 2009). This requires a high degree of spectral resolution in the SWIR, which is not available from Landsat nor from any current sensor that provides global coverage. Suitable narrow‐band satellite imagery is, however, available from research‐scale super‐spectral sensors (e.g., WorldView3; Satellite Imaging Corporation, n.d.) and imaging spectrometers (e.g., the Earth Surface Mineral Dust Source Investigation imaging spectrometer aboard the International Space Station [EMIT]; Connelly et al., 2021; Thompson et al., 2024; and the Italian *PRecursore IperSpettrale della Missione Applicativa* spaceborne imaging spectrometer [PRISMA]; Cogliati et al., 2021), and can be processed to accurately measure fCRC using methods described in detail in this manuscript.

### 1.6 Deriving remote sensing input for decision support tools and watershed models

The Precision Sustainable Agriculture Network (https://www.precisionsustainableag.org/), in collaboration with the LCB LTAR, conducts research focused on developing decision support tools to improve adoption and management of cover crops at the field scale. An example is the cover crop nitrogen calculator (CC‐NCALC; https://covercrop‐ncalc.org/). This is a web‐based decision support tool that provides geographically specific predictions of nitrogen release during cover crop decomposition to assist farmer decision‐making for field crop fertilization rates and timing (Thapa et al., 2022). The tool is based on the Crop Environment REsource Synthesis (CERES) nitrogen sub‐model and relies upon farmer‐reported cover crop biomass and forage quality analysis results for nitrogen as well as lignin, holocellulose, and non‐structural carbohydrate concentration (i.e., “carbon traits”). While the CC‐NCALC tool currently runs using farmer‐supplied input, this information is laborious and costly to collect. Accordingly, there is interest in developing remote sensing data products to parameterize the model, with the benefit of a quicker and easier application in the precision agriculture framework.

While satellite imagery can provide accurate measures of cover crop aboveground biomass, nitrogen content, and fractional cover, as well as crop residue cover, the methods are necessarily limited to measurement of the field surface. To extend analysis to transport of nutrients and carbon in the broader landscape, process‐based models such as the Soil and Water Assessment Tool (SWAT; Arnold et al., 1998) can be helpful to simulate the environmental impact of conservation practices. SWAT is a spatially explicit watershed model with detailed representation of crop growth and management, evapotranspiration, soil moisture, runoff, groundwater, soil nutrient cycling, river routing, and water quality processes (Neitsch et al., 2011). Recently, the SWAT‐Carbon model was developed by linking terrestrial biogeochemical algorithms based on CENTURY (Parton et al., 1994), Environmental Policy Integrated Climate (Izaurralde et al., 2006), and aquatic biogeochemical algorithms from QUAL2K (Chapra et al., 2003) and CE‐QUAL‐W2 (Cole & Wells, 2006). The SWAT‐Carbon model has been verified with field data for simulating soil carbon and nitrogen dynamics (Liang et al., 2022, 2023; Zhang et al., 2013) and riverine carbon and nitrogen fluxes (Qi et al., 2020; Wang et al., 2020). With these improvements, the SWAT‐Carbon model can simultaneously assess multidimensional environmental impacts of cover crops, including greenhouse gas emissions, soil health, and water quantity and quality.

### 1.7 Objectives

To measure the environmental impact of agricultural conservation practices, our work has focused on satellite‐based analysis of the three components of agricultural land cover: green vegetation (e.g., cover crops), NPV (e.g., crop residue), and exposed mineral soil. Applied in the wintertime between the summer cropping periods, the remote sensing methods are challenged by low sun angles, wet conditions, and fractional cover of endmembers (mixed pixels) at low levels of biomass. Our techniques have been developed to address these challenges, with the goal of mapping conservation performance at the field and landscape scale to inform adaptive management of agricultural conservation programs. This manuscript provides an overview of remote sensing research findings and applications associated with the LCB LTAR, documenting a variety of previously published outcomes (Table 1), with update and expansion of the techniques using additional unpublished data and analyses as appropriate.

**TABLE 1:** Select remote sensing publications from 10 years of activity at the USDA‐ARS Lower Chesapeake Long‐Term Agricultural Research network (LTAR) program.

Subject	Focus	Sensor(s)	Authors and year	Link
Cover crops	Mapping cover crop performance in Maryland (biomass and nitrogen uptake)	SPOT (Satellite pour l'Observation de la Terre) multispectral satellite	Hively et al. (2009)	https://doi.org/10.2489/jswc.64.5.303
	Mapping cover crop performance in Pennsylvania (wintertime greenness)	SPOT	Hively, Duiker, et al. (2015)	https://doi.org/10.2489/jswc.70.6.340
	Multispectral indices measuring cover crop performance (biomass and fractional cover)	Landsat, SPOT, WV‐2	Prabhakara et al. (2015)	https://doi.org/10.1016/j.jag.2015.03.002
	Within‐Season Emergence algorithm using HLS imagery (BARC field crops)	HLS	Gao, Anderson, Daughtry, et al. (2020)	https://doi.org/10.1016/j.rse.2020.111752
	Within‐Season Termination algorithm using VENuS imagery (BARC fields)	VENuS, Sentinel‐2	Gao, Anderson, and Hively (2020)	https://doi.org/10.3390/rs12213524
	Mapping cover crop performance in Maryland (NASA DEVELOP)	Landsat, Sentinel‐2	Thieme et al. (2020)	https://doi.org/10.1016/j.rse.2020.111943
	Optical and Radar sensors measuring cover crop performance (biomass)	Sentinel‐2, Sentinel‐1	Jennewein, Lamb, Hively, Thieme, Thapa, et al. (2022)	https://doi.org/10.3390/rs14092077
	Mapping cover crop performance throughout Maryland (biomass and nitrogen uptake)	Landsat, SPOT	Thieme et al. (2023)	https://doi.org/10.1002/agj2.21207
	Within‐Season Termination algorithm using HLS imagery (BARC, MD)	HLS	Gao et al. (2023)	https://doi.org/10.1016/j.srs.2022.100073
	Multispectral indices measuring cover crop performance (biomass, fractional cover)	Landsat, SPOT, WV‐2	Thieme et al. (2024)	https://doi.org/10.3390/s24072339
	Overview of remote sensing for Maryland cover crop program management	HLS	Hively et al. (2024)	https://doi.org/10.1109/IGARSS53475.2024.10640620
	Spectrometry to measure cover crop forage quality and carbon traits	PRISMA, ASD	Jennewein et al. (2024)	https://doi.org/10.1007/s11119‐024‐10159‐4
	Red edge indices for measuring cover crop performance (nitrogen content)	Sentinel‐2	Thieme et al. (2025)	https://doi.org/10.1002/agj2.70011
Crop residue	Narrow‐band and broad‐band SWIR indices for crop residue mapping	Landsat, Hyperion	Daughtry et al. (2006)	https://doi.org/10.1016/j.still.2005.11.013
	Moisture adjustments to narrow‐band SWIR indices for crop residue mapping	ASD	Daughtry and Hunt (2008)	https://doi.org/10.1016/j.rse.2007.08.006
	Narrow‐band SWIR indices for crop residue mapping	Aster	Serbin et al. (2009)	https://doi.org/10.3390/rs1040971
	Moisture adjustments to narrow‐band SWIR indices for crop residue mapping	ASD	Quemada and Daughtry (2016)	https://doi.org/10.3390/rs8080660
	Landsat and WorldView 3 for crop residue mapping	Landsat, WV‐3	Daughtry et al. (2018)	https://doi.org/10.1109/IGARSS.2018.8519473
	Narrow‐band SWIR indices for crop residue mapping	WV‐3	Hively et al. (2018)	https://doi.org/10.3390/rs10101657
	Moisture adjustments to narrow‐band SWIR indices for crop residue mapping	WV‐3	Quemada et al. (2018)	https://doi.org/10.1016/j.rse.2017.12.012
	Landsat and WorldView 3 for crop residue mapping	Landsat, WV‐3	Hively et al. (2019)	https://doi.org/10.3390/rs11161857
	Landsat Next band specification development	ASD	Hively et al. (2021a)	https://doi.org/10.3390/rs13183718
	Landsat Next band specification development	ASD	Lamb et al. (2022)	https://doi.org/10.3390/rs14236128
	Landsat Next band specification development	ASD	Dennison et al. (2023)	https://doi.org/10.1016/j.rse.2023.113715
	Landsat Next band specification development	ASD	Lamb et al. (2024)	https://doi.org/10.1109/IGARSS53475.2024.10640841
	Narrow‐band versus broad‐band SWIR spectral indices from WorldView3 imagery	WV‐3	Lamb et al. (2025)	https://doi.org/10.1016/j.still.2025.106524
Modeling	SWAT modeling of winter cover crops (nitrogen leaching)	n/a	Yeo et al. (2013)	https://doi.org/10.5194/hessd‐10‐14229‐2013
	SWAT modeling of winter cover crops (nitrogen leaching)	n/a	Lee et al. (2016)	https://doi.org/10.1371/journal.pone.0157637
	SWAT modeling of winter cover crops (nitrogen leaching)	n/a	Lee et al. (2017)	https://doi.org/10.13031/trans.12390
	SWAT modeling of winter cover crops (nitrogen leaching)	n/a	Lee, Yeo, et al. (2018)	https://doi.org/10.5194/hess‐22‐689‐2018
	SWAT modeling of winter cover crops (nitrogen leaching)	Landsat	Hively, Lee, et al. (2020)	https://doi.org/10.2489/jswc.75.3.362
	DSSAT model parameters from remote sensing	Sentinel‐2, Planet	Akumaga et al. (2023)	https://doi.org/10.3390/agronomy13061540
	SWAT modeling of winter cover crops (nitrogen leaching and carbon)	n/a	Zhang et al. (2024)	https://doi.org/10.1016/j.jenvman.2024.123104
LTAR site	Lower Chesapeake Bay LTAR weather station network	n/a	Cosh and McKee (2023)	https://doi.org/10.15482/USDA.ADC/1528550
	Lower Chesapeake Bay LTAR Common Experiment description	n/a	Bagley et al. (2024)	https://doi.org/10.1002/jeq2.20650

Abbreviations: ASD, Analytical spectral devices; BARC, Beltsville Agricultural Research Center; HLS, Harmonized Landsat Sentinel‐2; n/a, not applicable; NASA, US National Aeronautics and Space Administration; PRISMA, *PRecursore IperSpettrale della Missione Applicativa* spaceborne imaging spectrometer; SPOT, *Satellite pour l'Observation de la Terre* multispectral satellite; SWIR, shortwave infrared; SWAT, Soil and Water Assessment Tool.

## 2 MATERIALS AND METHODS

### 2.1 Study area

**FIGURE 1:**
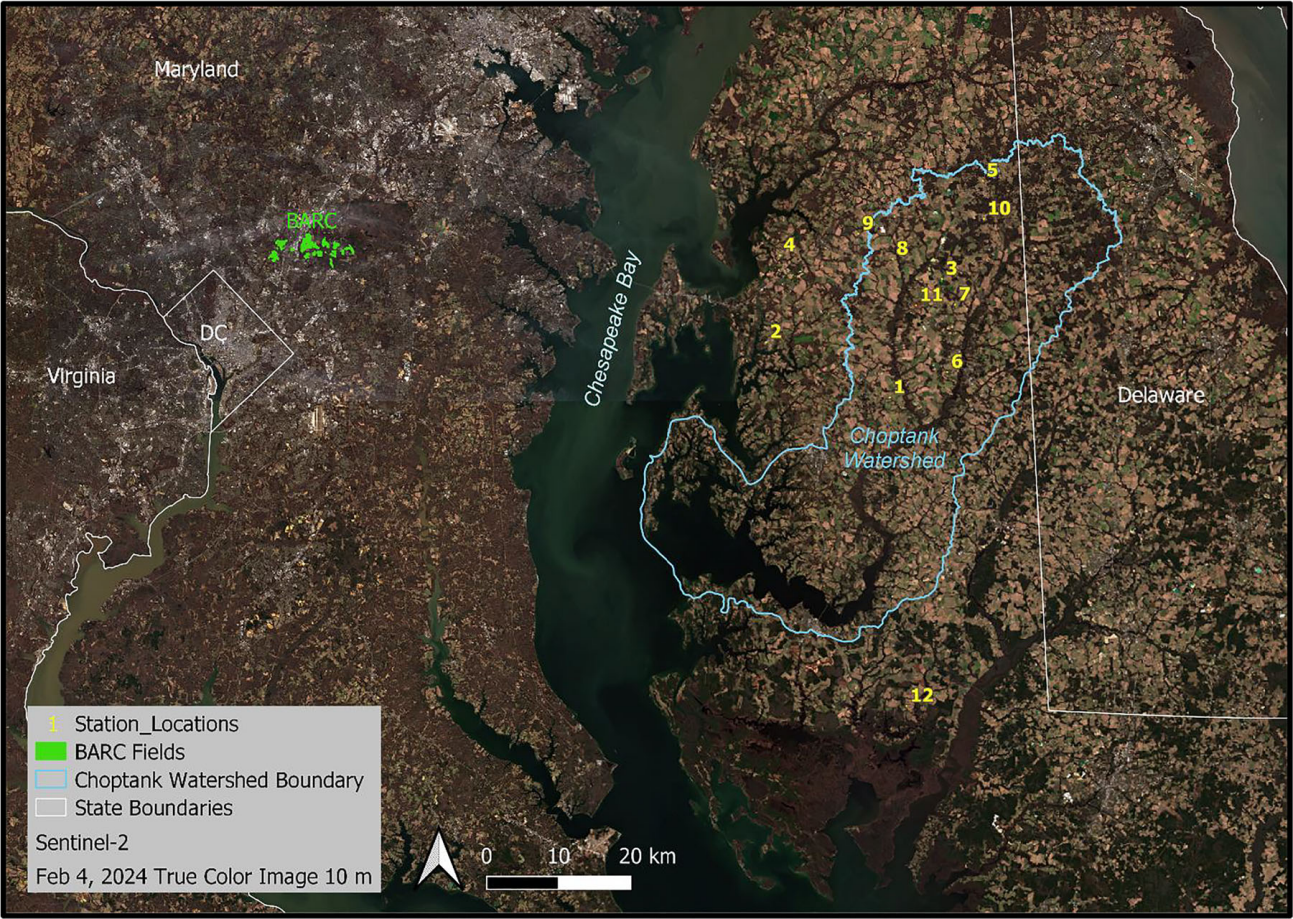
Study area map showing a portion of the Delmarva Peninsula containing the Choptank River watershed (light blue) and Long Term Agroecosystem Research weather stations (yellow numbers; Cosh & McKee, 2023), as well as the Beltsville Agricultural Research Center (BARC). Basemap: Sentinel‐2 satellite imagery (10 m) from February 4, 2024.

The core research area of the LCB LTAR is the Mid‐Atlantic Coastal Plain region of the United States, with field data collection (2006–present) focused at the USDA‐ARS BARC and on working farms near or within the Choptank River watershed, on the Delmarva Peninsula, Maryland (Figure 1). Climate in the region is humid subtropical with 12°C average temperature, 111 cm average rainfall, and an average of 18 winter days below freezing (Cosh & McKee, 2023; Hively et al., 2011). Soils are generally silt loams (BARC) or silt loams and sandy loams (Delmarva). Predominant crop sequences (Lee et al., 2017) are corn‐soy and corn‐soy‐double crop winter wheat/soy (common on grain farms and farms with associated broiler poultry production, more predominant in the southern extent of the Delmarva) and also corn silage/winter rye silage on dairy farms (more commonly found at the northern extent of the Delmarva). Winter cover crops are often planted in the fallow season between summer cash crops, with greatest frequency after corn due to earlier harvest dates and lowest frequency after double‐crop soy, corresponding to later harvest dates. The remote sensing tools developed in this region have also been extended to applications in Delaware, Pennsylvania, and Missouri.

The LCB LTAR has established 12 on‐farm weather stations across the Delmarva area (Figure 1), operated since summer 2014. Each weather station measures soil moisture and soil temperature at four depths (5, 10, 20, and 50 cm), as well as precipitation and air temperature. Five of the stations also measure incoming photosynthetically active radiation (PAR). The data are recorded every 60 min. Several of the weather stations have been decommissioned or relocated over time. High‐latency data are available at Cosh and McKee (2023), and lower latency data are available on request. Growing degree days (GDD) are calculated from the daily average air temperature data using a base temperature for cover crop growth of 4°C; frost degree days are calculated as degrees below 0°C.

### 2.2 Satellite remote sensing of winter cover crop growth

#### 2.2.1 Incentive program field boundaries and agronomic data

Privacy protection agreements with collaborating conservation stakeholders in Maryland, Delaware, Pennsylvania, and Missouri have supported access to annual cover crop cost‐share implementation datasets. These data include polygon boundaries (Maryland, Missouri, and Pennsylvania) or centroids (Delaware) for each field enrolled in cover crop cost‐share programs, along with associated field‐specific agronomic management information (e.g., cover crop species, planting date and method, termination date and method, and previous crop). Extensive interaction with conservation stakeholders is required to obtain, check, and standardize the enrollment datasets, as well as to identify stakeholder management objectives and environmental endpoints.

Early collaboration with the MDA supported the development of online tools that are now routinely used by staff at each Maryland soil conservation district to digitize cover crop field boundaries and record associated agronomic management data for each year of program enrollment. Management information includes cover crop species, planting date and method, previous crop, manure application, and termination date and method for each field, as reported by the enrolled farmers. This information is compiled into a database used by the MDA for annual MACS cover crop program management including verification protocols. The privacy‐protected dataset is provided to our research team by MDA each December following fall enrollment certification (>25,000 fields per year), and we use the data to support remote sensing analyses, reporting results in a manner that maintains farmer confidentiality.

#### 2.2.2 Satellite imagery data extraction

The main data source that we use to evaluate cover crop growth curves and environmental performance at the field and landscape scale is HLS satellite imagery, which provides no‐cost multispectral reflectance data at 30 m resolution. The harmonization, which accounts for differences in sensor band characteristics between Landsat 8‐9 and Sentinel‐2 satellites, allows interchangeable use of both datasets, achieving a 3–5 day return frequency with global coverage (Claverie et al., 2018). The imagery is downloaded from NASA's Earth Data Distributed Active Archive Center (https://search.earthdata.nasa.gov) as Level 2A surface reflectance, to which the native cloud mask is applied (values 0, 64, and 128 are kept, corresponding to clear conditions, low, and moderate aerosol levels, respectively), after which the NDVI is calculated as NDVI = (NIR − Red)/(NIR + Red), where NIR and Red are the near‐infrared and red reflectance bands, respectively. While additional multispectral indices, including red edge and SWIR wavelengths, can provide utility in measuring nitrogen content and extending the range of analysis prior to index saturation, our research has demonstrated that NDVI is consistently a top performer in winter cover crop multispectral applications (Jennewein et al., 2022; Prabhakara et al., 2015) and is therefore favored for reasons of parsimony and its compatibility with both Landsat and Sentinel‐2 sensors.

For each field enrolled in the MACS cover crop program, field boundary polygons are buffered inward by −30 m to remove mixed‐pixel effects associated with field edges, and the temporal sequence of spatial median NDVI values for each available HLS image over a given cover crop season is extracted into a tabular dataset. A flexfit algorithm (Gao, Anderson, Daughtry, et al., 2020) using a modified Savitzky–Golay filter is then applied to derive interpolated daily NDVI values for each field from early fall (September 1) to late spring (May 31), encompassing the winter cover crop growing season in the Mid‐Atlantic region. A growth curve is subsequently graphed for each field, including both farmer‐reported agronomic information and HLS‐derived measurements.

**FIGURE 2:**
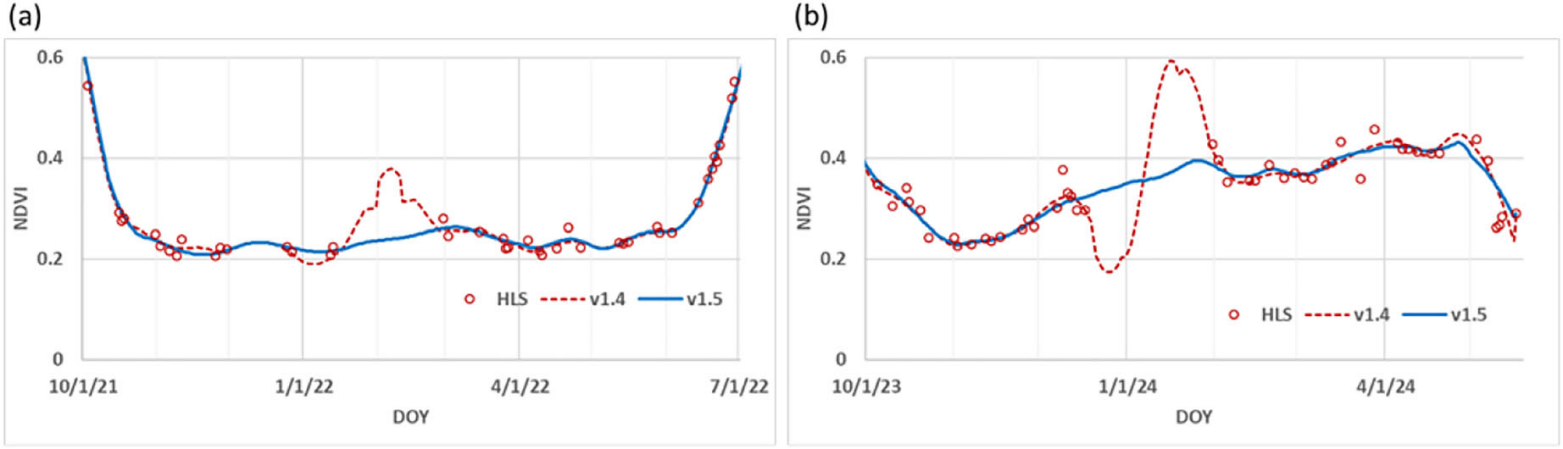
Improvements to the flexfit v.1.4 interpolation for gaps in the imagery sequence are achieved by implementing an initial 3‐day smoothing prior to the flexfit (a) and reducing the order of the polynomial function within large data gaps (b). As shown in these examples, flexfit version 1.5 (blue line) generated a better fitting normalized difference vegetation index (NDVI) daily time series than version 1.4 (dashed red line) using an identical input dataset of Harmonized Landsat Sentinel‐2 (HLS) observations. DOY, day of year.

The flexfit algorithm requires a minimum number of clear observations within the moving window to avoid erratic predictions in times of extended cloud or snow cover. In the original version 1.4 (Gao, Anderson, Daughtry, et al., 2020), we defined the minimum number of observations as six within a moving window of ± 75 days. However, we continued to observe erratic predictions under some conditions and have now updated the flexfit algorithm to include two new restrictions in the current version 1.5. The first is to reduce the order of polynomial fitting (from third to second order) in the Savitzky–Golay filter for large temporal gaps, defined as less than six clear observations within ±30 days. This revision improves interpolation for a large temporal gap by avoiding the over‐fitting from a higher order polynomial function (Figure 2a). A second improvement addresses the problem of uneven distribution of observations when, in some cases, many clear observations may be available for a short period. The interpolation is very sensitive to the quality of the lumped observations, and a small directional variation from neighboring days can have a large impact on the broader interpolation (v. 1.4; Figure 2a). To address this problem, the revised algorithm (v. 1.5) uses a 3‐day interpolation to smooth small variations among closely neighboring observations, while observations with >3‐day incidence interval remain unchanged. The resulting data are then interpolated to calculate an improved daily NDVI time series (Figure 2b).

For very small agricultural fields (typically <1.6 ha [<4 acres]), the 30‐m pixel size of HLS imagery is often too large to derive a clear measurement of field conditions without inclusion of mixed pixels along the field boundary. In these cases, we have explored the use of PlanetScope Superdove imagery (Planet Team, 2024). Downloaded in November 2024, the recent Planet surface reflectance products now include improved cross‐sensor calibration among the various Superdove platforms (Keeley Roth, oral communication, December 2024). To compare the relative accuracy of PlanetScope to HLS retrievals, we downloaded a multi‐temporal sequence of images from both data sources, covering a subset of enrolled cover crop fields on the Delmarva Peninsula (*n* = 2700) from September 1, 2023, through June 31, 2024. We computed field median NDVI values from Superdove imagery and compared these to HLS‐derived values for the same dates using simple linear regression. Next, we ran Superdove extractions through the flexfit algorithm to obtain daily interpolated NDVI values per field and compared these to HLS flexfit over the 2023–2024 cover crop season.

#### 2.2.3 Identifying cover crop emergence, performance, and termination

Critical inflection points are identified in each HLS‐derived daily NDVI growth curve using the Within‐Season Emergence (WISE) and Within‐Season Termination (WIST) algorithms (Gao, Anderson, & Hively, 2020; Gao et al., 2023). These algorithms can be tuned to detect stronger versus weaker green‐ups and terminations based upon calculations of event momentum and magnitude of change, with corresponding tradeoffs in errors of omission and commission. While green‐up dates are unconstrained, termination dates are limited to March 1–May 31 to avoid false positives resulting from mid‐winter downturns associated with cover crop chlorosis and dormancy under freezing conditions. This constraint is relaxed for fields known to be planted to winter‐kill species (e.g., monoculture forage radish [*Raphanus sativus* L.] and oats [*Avena sativa* L.]), and for fields subject to mid‐winter grazing.

Seasonal performance maxima (NDVI value and date observed) for each field are extracted from the HLS‐derived growth curves for the winter (November 15–February 15) and spring (February 15–May 30) seasons, constrained between observed green‐up and termination dates. The winter value is indicative of growth obtained by the cover crop prior to mid‐winter freezing weather and the hydrologically active period when significant nitrate leaching occurs. The spring value represents additional growth achieved during warm springtime conditions.

The seasonal NDVI maxima are converted to estimated fractional green cover (%, *n* = 711, *R* ^2^ = 0.62–68, RMSE = 13.1%–14.6%; Thieme et al., 2020) and/or estimated aboveground biomass (kg ha^−1^, *n* > 700, *R* ^2^ = 0.57–0.77, RMSE = 0.40–0.83 ln kg ha^−1^; Thieme et al., 2023) depending on season and species, through correlation with field data from a decade of extensive on‐farm sampling conducted using quadrats and cameras. Field sampling methods are described in detail in Thieme et al. (2023) and Jennewein et al. (2024). From 2005 to 2012, field sampling also included determination of soil inorganic‐N concentration in the top 30 cm (2 M KCl extraction with Lachat analysis; inorganic‐N = nitrate‐N + ammonium‐N; Hively et al., 2009).

### 2.3 Satellite remote sensing of crop residue cover

#### 2.3.1 Narrow‐band SWIR satellite imagery and field data collection

To measure lignocellulose absorption, we have acquired and analyzed narrow‐band super‐spectral satellite imagery (WorldView‐3; Satellite Imaging Corporation, n.d.), hyperspectral satellite imagery (PRISMA and EMIT), and laboratory spectral measurements (Analytical Spectral Devices [ASD] ViewSpec Pro 4; Lamb et al., 2022, 2024, 2025). Each spring season since 2015, our research team has sampled in situ fCRC on a collaborating farm on the Delmarva Peninsula that uses a broad range of tillage practices on different fields, including plow tillage (used to prepare clean fields for cucumber production), reduced tillage (disking and turbo‐till), and long‐term no‐till. Each year, this produces a broad range of on‐farm fCRC measurements (0%–98%, *n* = 895, for 2015–2022) that are used to calibrate and validate satellite analyses and develop algorithms for mapping crop residue cover in the surrounding landscape. Methods used to acquire and process satellite imagery (Hively et al., 2018, 2019; Jennewein et al., 2024; Lamb et al., 2025; Thieme et al., 2023), laboratory spectral measurements and analysis (Dennison et al., 2023; Hively et al., 2021a; Lamb et al., 2022; Quemada & Daughtry, 2016), and field calibration data (Hively et al., 2018; Lamb et al., 2025) are described in the associated references.

## 3 RESULTS AND DISCUSSION

### 3.1 Winter cover crops

#### 3.1.1 Weather station data

Wintertime greenness (presence of living vegetative ground cover) is strongly associated with the warmth of wintertime growing conditions, with warmer conditions promoting more abundant growth of cover crops, weeds, and winter small grain crops. Multi‐year remote sensing trend analysis of cover crops must therefore establish that satellite imagery is collected at times of similar GDD accumulation (e.g., Hively, Duiker, et al., 2015) or must seek to control for variation in wintertime climate using a measure such as wintertime accumulated GDD (e.g., Hively, Lee, et al., 2020). In the Choptank River watershed, the LTAR weather stations have recorded increasingly warm winters in recent years (Table 2) with five of the warmest winters on record occurring in the past 6 years.

**TABLE 2:** Accumulated growing degree days (GDD), frost degree days (FDD), and average maximum winter normalized difference vegetation index (NDVI, before March 1) for cover crop wheat fields with conventional planting method (drill + light tillage), calculated using data from the CHOP‐1 weather station.

Winter cover crop season	Accumulated GDD		Accumulated FDD		Number of fields		Max winter NDVI	
	October 15–March 1	November 5–March 1	October 15–March 1	November 5–March 1	Early planted	Late planted	Early planted	Late planted
2017–2018	598	297	298	298	114	172	0.67	0.48
2018–2019	458	226	219	219	105	251	0.54	0.40
2019–2020	534	271	122	122	162	261	0.60	0.48
2020–2021	572	292	173	173	122	233	0.60	0.40
2021–2022	612	300	221	221	85	271	0.64	0.52
2022–2023	624	376	127	127	74	207	0.68	0.56

*Note*: Early‐planted fields are planted by October 14, while late‐planted fields are planted between October 15 and November 5.

**FIGURE 3:**
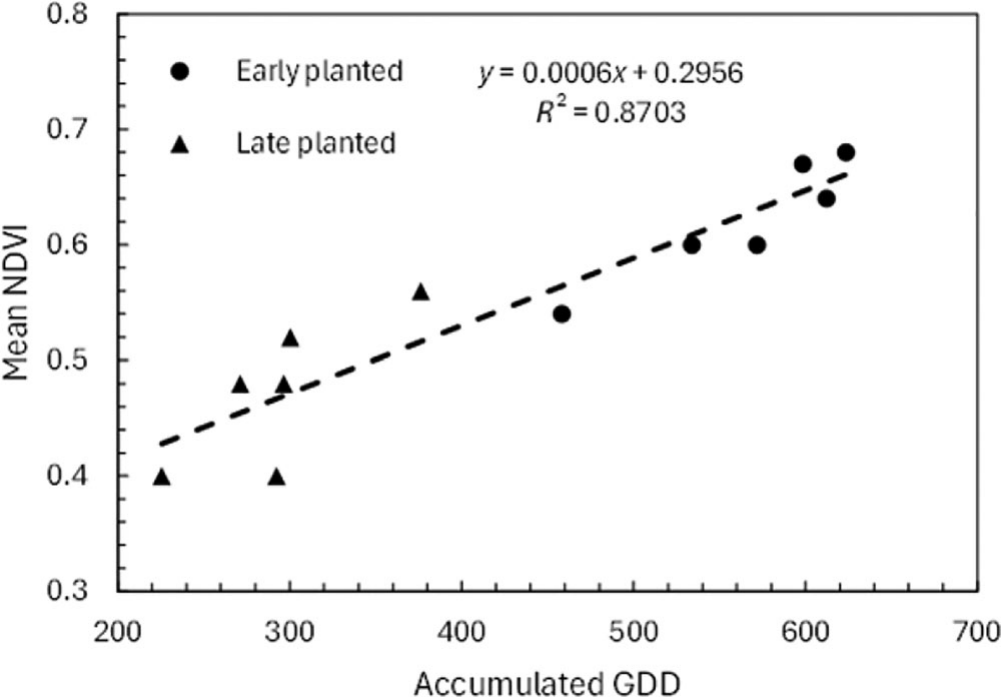
Annual average of wintertime maximum normalized difference vegetation index (NDVI) values for all drilled wheat cover crop fields enrolled in the Maryland Agricultural Cost Share (MACS) program for fields planted early (before October 15) and late (October 15–November 5), with correlation to accumulated annual wintertime growing degree days (GDD) prior to March 1, with GDD summation beginning October 15 and November 5 for the early‐ and late‐planting date categories, respectively (data from Table 2).

The annual average wintertime maximum NDVI was calculated for all drilled winter wheat cover crop fields planted early (before October 15) versus planted late (October 15–November 5). When the resulting values (Table 1) were compared to accumulated wintertime GDD beginning October 15 and November 5, respectively, a strong relationship was detected (*R* ^2^ = 0.87), demonstrating that year‐to‐year variation in cover crop performance was strongly related to the accumulated warmth of each wintertime growing season (Figure 3). This analysis was limited to drilled wheat fields to reduce variation attributable to planting methods and cover crop species.

**FIGURE 4:**
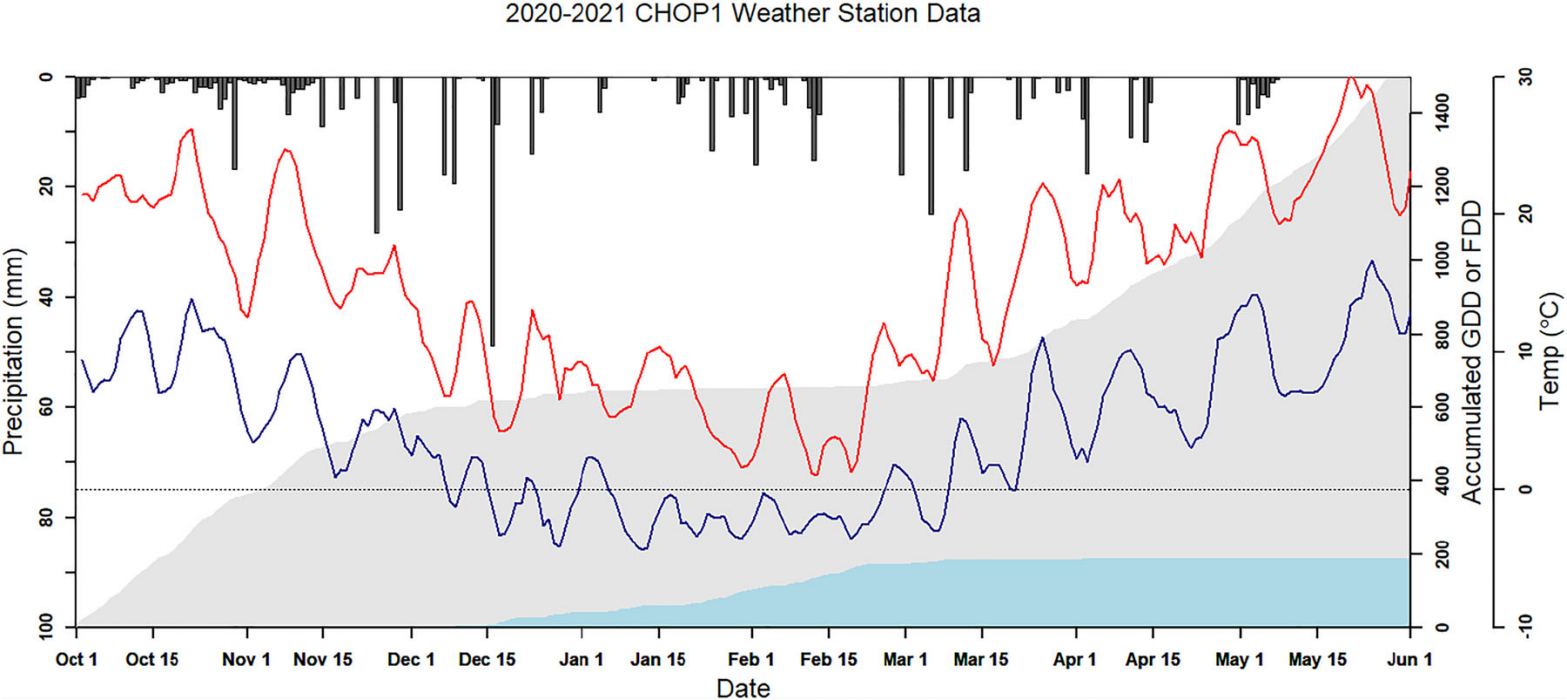
Data from the CHOP1 weather station showing daily maximum (red line) and minimum (blue line) temperatures (°C), accumulated growing degree days (GDD, gray area), accumulated frost degree days (FDD, light blue area), and precipitation (dark gray bars) for the 2020–2021 cover crop season.

Our research has also documented that wintertime freezing periods (Figure 4) are associated with onset of chlorosis and dormancy in winter cover crops, with a resulting decrease in NDVI relative to accumulated aboveground biomass. This has required the development of separate calibration equations to derive aboveground biomass from NDVI for the early (pre‐frost) winter, the cold mid‐winter, and warmer spring conditions (Prabhakara et al., 2015; Thieme et al., 2023, 2020). The springtime calibration curves for cereal cover crop biomass were somewhat species dependent, likely related to differences in leaf angle distribution as well as species‐specific sensitivity to frost damage (e.g., greater amount of springtime non‐photosynthetic frost‐impacted biomass in barley relative to the other species). Work is ongoing to accurately characterize vegetation index calibration curves for different time periods, species, and species mixes, as well as accounting for regional variability in background soil color.

#### 3.1.2 Characterization of cover crop growth curves using HLS and PlanetScope imagery

**FIGURE 5:**
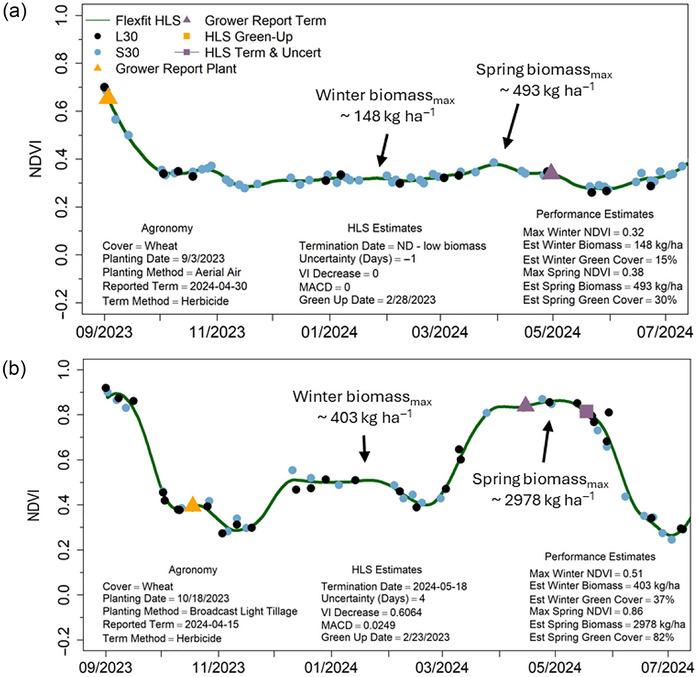
Example growth curve plots derived from field‐level median normalized difference vegetation index (NDVI) values retrieved from 30 m resolution Harmonized Landsat and Sentinel‐2 multispectral satellite imagery (HLS) imagery with points indicating Landsat (L30) and Sentinel‐2 (S30) imagery acquisition dates: (a) represents a poor‐performing field and (b) a high‐performing field. Observed dates of cover crop emergence (HLS Green Up Date) are associated with farmer‐reported cover crop planting dates (Agronomy Planting Date), while observed HLS Termination Dates and calculated Uncertainty are associated with farmer‐reported cover crop termination dates (Agronomy Reported Term, purple triangles).

**FIGURE 6:**
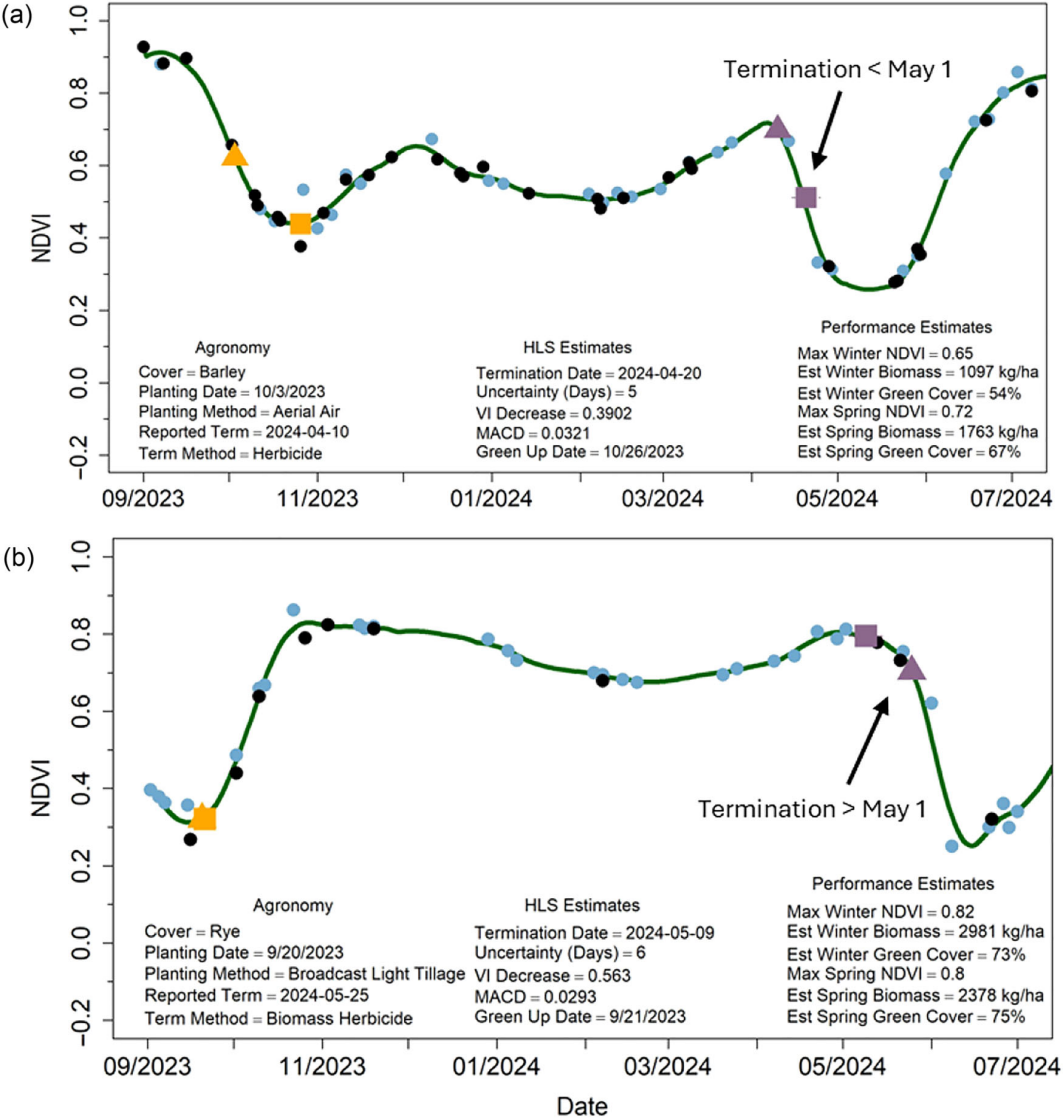
Example growth curve plots derived from field‐level median normalized difference vegetation index (NDVI) values retrieved from 30 m resolution Harmonized Landsat and Sentinel‐2 multispectral satellite imagery (HLS) imagery with points indicating Landsat (L30) and Sentinel‐2 (S30) imagery acquisition dates: (a) represents a regular termination date field with good performance and (b) late termination of a cover crop. Observed dates of cover crop emergence (HLS Green Up Date) are associated with farmer‐reported cover crop planting dates (Agronomy Planting Date), while observed HLS Termination Dates and calculated Uncertainty are associated with farmer‐reported cover crop termination dates (Agronomy Reported Term, purple triangles).

Updates to the flexfit algorithm have improved our ability to accurately monitor cover crop growth curves, phenology, and performance. Example graphs derived from HLS imagery demonstrate cover crop fields with varied levels of performance (Figure 5), ranging from poor (a) to high biomass (b), as well as late‐termination dates (c) and early‐termination dates (d) (Figure 6).

The accuracy of cover crop green‐up and termination dates derived from the WISE and WIST algorithms has been documented in Gao, Anderson, and Hively (2020) and Gao et al. (2023), respectively. For the operational springtime termination assessment for MDA, cover crop fields are divided into various categories (e.g., high and low biomass fields with termination dates matching or not matching farmer‐reported eradication dates, or no termination detected) (Hively et al., 2024). For the 2023–2024 cover crop season, remote sensing analysis detected termination on nearly all fields (*n* = 24,020; ∼98%) while identifying a small subset of fields for which no termination was detected (*n* = 602; ∼0.20%). The latter category was flagged for site visits by field staff, who found some fields where cover crops were not present or had erroneously been grown to harvest (payment was subsequently denied) and others where summer crops were present and the remote sensing analysis had failed due to planting green, weediness, imagery gaps, or other factors.

**FIGURE 7:**
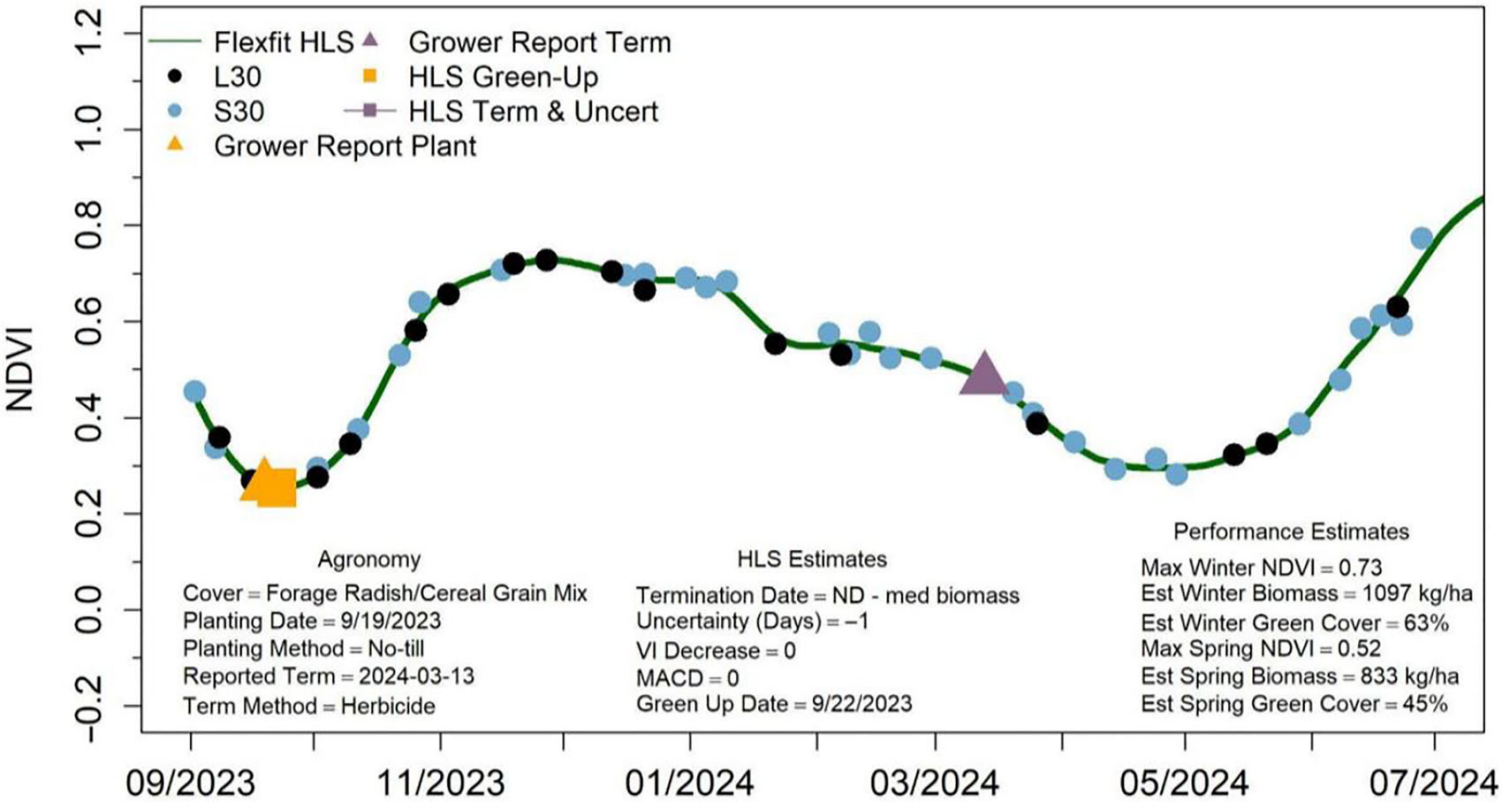
Example cover crop growth curve with no termination detected from the within‐season termination (WIST) algorithm despite a clear downward trend, due to the downturn inflection point occurring in November, prior to the considered springtime termination window. Points indicate Landsat (L30) and Sentinel‐2 (S30) imagery acquisition dates. Observed dates of cover crop emergence (HLS Green Up Date) are associated with farmer‐reported cover crop planting dates (Grower Reported Plant), while observed termination dates and calculated uncertainty (HLS Term & Uncert) are associated with farmer‐reported cover crop termination dates (Agronomy Reported Term, purple triangle). HLS, Harmonized Landsat Sentinel‐2.

In recent efforts, several opportunities for continued improvement of the WIST algorithm have been identified. In cases where the maximum NDVI value occurred in the winter months (prior to March 1), followed by a slow reduction in NDVI through springtime, WIST does not detect a termination because the inflection point occurred prior to the considered springtime date range (Figure 7). Additionally, two outputs are provided by WIST, one affiliated with the largest downturn in NDVI and one associated with the first observed downturn in NDVI starting after the first day of the calendar year. We observed that in some cases, the first observed downturn more closely matched grower reported terminations, while in other cases, the largest NDVI downturn was more accurate. These issues appear to be situational, requiring field‐specific consideration, but future efforts could investigate which version of WIST output is most appropriate for a given species and field management operation.

**FIGURE 8:**
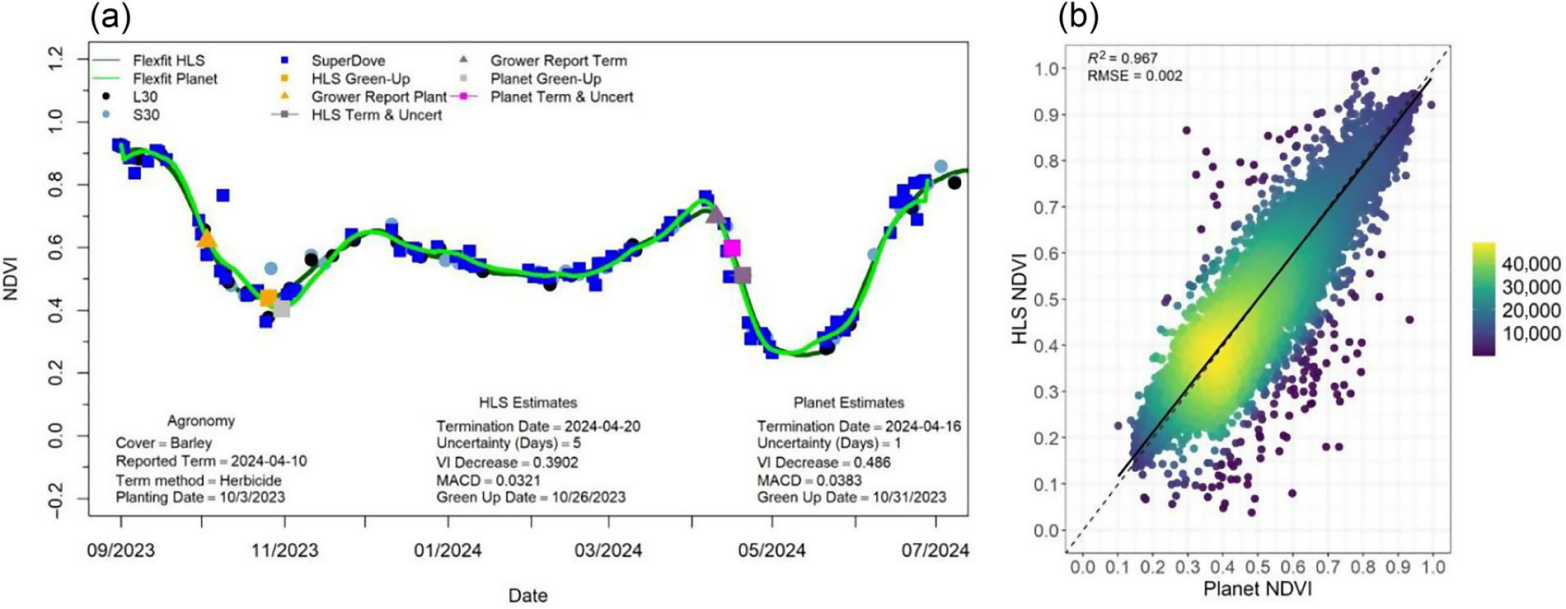
(a) Example growth curve plot depicting flexfit v. 1.5, within‐season emergence (WISE), and within‐season termination (WIST) estimates for both Harmonized Landsat and Sentinel‐2 multispectral satellite imagery (HLS) and PlanetScope Superdove imagery streams, and (b) scatterplot comparing field‐level median normalized difference vegetation index (NDVI) values for HLS and PlanetScope Superdove imagery where color indicates the density of points.

While HLS is a well‐calibrated and consistent satellite product (Claverie et al., 2018), the PlanetScope dataset used for analysis of small and/or strip‐cropped fields relies upon a fleet of small satellites, which has historically shown issues with cross‐calibration among sensors. Recent improvements to PlanetScope data processing have helped to address this concern (Keeley Roth, oral communication, December 2024). A comparison of flexfit growth curves calculated using HLS and using the current (November 2024) PlanetScope surface reflectance product demonstrated that resulting NDVI values are now fairly interoperable (Figure 8b; *R* ^2^ = 0.97, RMSE = 0.002). A visual inspection of the flexfit curves (Figure 8a) demonstrates good agreement where multitemporal retrievals of NDVI are largely consistent between image sources. We observed a few cases with >0.15 NDVI difference between HLS and Superdove imagery on the same day with the same field, but this occurred in only a small subset of observations (∼0.5%) and may be related to cloud masking algorithms. These results indicate that when HLS imagery (30 m) is too spatially coarse for analysis (small, strip‐cropped, or irregularly shaped agricultural fields, as well as plot‐level research studies), the smaller pixel size (3.7 m) and daily return frequency of PlanetScope imagery can now be successfully used to monitor and report NDVI‐related performance metrics as well as critical field management information (emergence and termination dates). Future work could statistically compare PlanetScope and HLS‐derived WIST and WISE estimates for the same set of fields and in different regions to further quantify accuracy and determine any adjustment to critical phenological point determinations.

#### 3.1.3 Cover crop performance

For each year of MACS enrollment data (2015–present), seasonal maximum NDVI values were used to calculate cover crop performance for each cover cropped field (∼25,000 per year). Findings demonstrate that earlier planting dates (before October 15) and better seed‐to‐soil contact (drilled) are associated with increased cover crop performance, and that later planting dates, broadcast or aerial seeding methods, and wheat species are associated with decreased cover crop performance (Hively et al., 2009; Thieme et al., 2023). In Maryland, from 2008 to 2017, 68.1% of cover crop fields were wheat and 34.7% were late‐planted wheat (Hively, Lee, et al., 2020), showing considerable opportunity for increasing cover crop performance by shifting toward earlier plantings of higher performing cover crop species. Thieme et al. (2023) also calculated the impact of delaying springtime termination until after May 1 (for which MACS paid an additional incentive payment). Results (2020–2021) showed an estimated average increase of 789 kg ha^−1^ (63%) of biomass, 15 kg ha^−1^ (48%) of nitrogen uptake, and 336 kg ha^−1^ (63%) of carbon associated with fields terminated after May 1 (*n* = 19,120) over fields that did not delay termination (*n* = 28,811). It is also possible to measure cover crop nitrogen content directly using satellites with red edge wavelengths. Thieme et al. (2025) demonstrated an *R* ^2^ of 0.748 and an RMSE of 13.1 kg ha^−1^ in prediction of cereal cover crop biomass nitrogen content using red edge indices derived from Sentinel 2 satellite imagery.

From 2005 to 2013, 1066 soil samples were collected along with on‐farm cover crop biomass on the Delmarva Peninsula in the fall, winter, and spring seasons (Table 3). Average soil inorganic‐N (0–20 cm, 2 M KCl extraction, nitrate‐N + ammonium‐N) was 31 kg ha^−1^ in fall and 17 kg ha^−1^ in winter with 89.5% of fields above 10 kg ha^−1^ in fall and 65.9% of fields above 10 kg ha^−1^ in winter. By wintertime, cover crops aboveground biomass had captured an average of 52% of total plant‐soil N (range 0.5%–97.4%), rising to 70% in the springtime.

**TABLE 3:** Description of seasonal sampling of enrolled cover crop fields on the Delmarva Peninsula from 2005 to 2013, including mean analytical results for soil samples and cover crop biomass samples (*n* = 1066).

			Fields with soil inorganic‐N above threshold value				Cover crop vegetation					
Season	*n*	Soil inorganic‐Nitrogen (kg ha^−1^)	10 kg ha^−1^		20 kg ha^−1^		Biomass (kg ha^−1^)	N concentration (%)	N content (kg ha^−1^)	Sum N in soil and biomass^a^ (kg ha^−1^)	Fraction N in soil (%)	Fraction N in vegetation (%)
			#	%	#	%						
Fall	209	31	187	89.5	117	56.0	124	3.8	5	36	87	13
Winter	469	17	309	65.9	132	28.1	605	3.1	19	36	48	52
Spring	388	14	163	42.0	93	24.0	1188	2.9	34	49	30	70

*Note*: Some fall samples (*n* = 137) did not have sufficient cover crop biomass to sample (just planted or newly emerged); nitrogen concentration values are from cover crop samples, while biomass × nitrogen content (N content) includes 0 values for fields with minimal cover crops. Biomass samples included some small grain crops (*n* = 50) that may have had fall fertilization, but most were traditional cover crops that were restricted from fall fertilization.

^a^ Sum N in soil and biomass is the total of soil inorganic‐N and vegetation N content.

**FIGURE 9:**
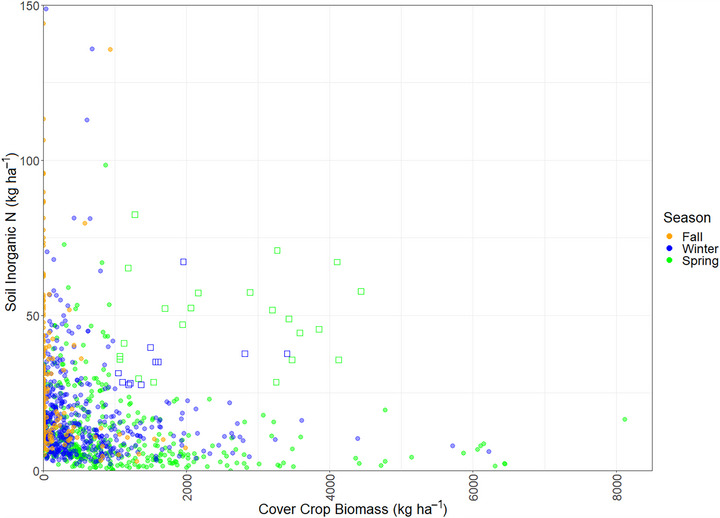
Cover crop biomass (kg ha^−1^) versus soil inorganic‐N (kg ha^−1^) with color indicating the sampling season (orange = fall, blue = winter, green = spring, *n* = 1066). Open squares indicate the 3% of samples (*n* = 33) with both high cover crop biomass (>1000 kg ha^−1^) and high soil N (>25 kg ha^−1^).

Wintertime soil nitrate‐N was inversely related with wintertime cover crop performance (Figure 9), with successful cover crops (>1000 kg ha^−1^ aboveground biomass) associated with lower amounts (<25 kg ha^−1^) of soil nitrate‐N in the top 30 cm, while soil nitrate‐N levels above 25 kg ha^−1^ were mainly associated with low‐performing cover crops (biomass < 1000 kg ha^−1^). Of the 3% of the fields with inorganic soil N levels above 25 kg ha^−1^ and cover crop biomass above 1000 kg ha^−1^ (*n* = 33, square symbols in Figure 9), the majority were rye or barley, three were chopped for silage in the spring, and 17 were harvested as a small grain crop, implying that the high observed soil nitrate‐N levels were the result of abundant manure application on dairy farms or springtime fertilization of cereal crops prior to sampling.

In the 2007–2008 and 2008–2009 cover crop seasons, repeat sampling of available soil inorganic‐N and cover crop aboveground biomass was conducted in the same set of fields in October (fall), December (winter), and April (spring) of each year. Fall sampling results (*n* = 209) showed considerable variability in soil inorganic‐N availability (5.3–204.8 kg ha^−1^). Wintertime biomass was insignificantly positively associated with fall soil inorganic‐N availability (*n* = 153 matched samples, *R* ^2 ^= 0.24, RMSE = 840.8 kg ha^−1^ biomass), indicating that while some cover crops were growth‐limited by low nitrogen availability, biomass showed considerable variability associated with planting date, planting method, species, and weather, among other factors, and fall availability of residual soil inorganic‐N was not the predominant driver of cover crop performance outcomes.

#### 3.1.4 Adaptive management of winter cover crop incentive programs

The satellite remote sensing analyses of enrolled cover crop fields that we have produced have been provided to MDA on an annual basis, with performance measures communicated at the end of each cover crop season and an operational termination‐date analysis conducted every 2 weeks throughout each springtime as HLS imagery and farmer‐reported cover crop terminations are updated (Hively et al., 2024). If farmer‐reported termination dates are similar to satellite‐derived dates, the MDA can use this information to verify termination and release payment for those fields. Field verification visits by conservation district staff can then be targeted to a subset of fields for which termination is not detected, resulting in substantial time savings. Some recent adaptive changes to the MACS incentive program (Maryland Department of Agriculture, 2024) that have resulted from multi‐year analyses linking performance to agronomy (e.g., Thieme et al., 2023) include increased incentives for triticale and a requirement that late‐planted cover crops be terminated after May 1 (without late‐termination payment).

### 3.2 Crop residue cover

#### 3.2.1 Lignocellulose index applications

Building on USDA‐ARS research that developed narrow‐band SWIR indices for crop residue characterization (CAI, SINDRI; Daughtry & Hunt, 2008; Daughtry et al., 2006; Nagler et al., 2000; Serbin et al., 2009), we have evaluated the utility of WorldView‐3 SWIR satellite imagery to map fCRC, comparing multiple WV3‐derived indices. Note that spectral data from WV3 can calculate the SINDRI but not CAI, because it lacks a band centered around 2040 nm. Analysis of data acquired in 2015 (Hively et al., 2018) showed significantly increased accuracy using the SINDRI (*R* ^2^ = 0.914, RMSE = 0.821) in comparison to the broadband Landsat‐based NDTI (*R* ^2^ = 0.788, RMSE = 0.127). A more recent publication has used a six‐year time series of Delmarva springtime WV3 imagery and field data collection to derive maps of fCRC on an annual basis, showing that under a broad range of surface moisture conditions and green vegetation cover the narrow‐band SINDRI (*R* ^2^ = 0.869, RMSE = 0.111) consistently outperformed HLS‐compatible indices NDTI (*R* ^2^ = 0.596, RMSE = 0.179) and SWIRA (*R* ^2^ = 0.617, RMSE = 0.176), although SWIRA was an improvement over NDTI (Lamb et al., 2025).

A limitation of WorldView‐3 analysis is the narrow swath of the imagery (12 km width). Therefore, WV‐3 analysis cannot be used to produce maps characterizing tillage intensity at a regional scale. For that purpose, we have developed techniques that use the WV3‐derived fCRC maps to calibrate a machine learning analysis of temporally proximal HLS imagery (Hively et al., 2019). Results show increased fCRC estimation performance using boosted regression trees applied to multiple Landsat 8 spectral bands and indices when compared to uncalibrated NDTI and other simple indices (92.1%–93.3% accuracy vs. 58.7%–83.7% accuracy, respectively). These results indicate an approach that can be used to accurately map residue cover at scale. Research currently underway is applying three machine learning techniques (neural networks, random forest, and extreme gradient boosting) to derive HLS‐based maps of fCRC for the Delmarva Peninsula using the six‐year time series of WV‐3‐derived map products for calibration. Future work will expand these techniques to imaging spectroscopy data sources including PRISMA and EMIT.

#### 3.2.2 Lignocellulose indices and impact of surface wetness

**FIGURE 10:**
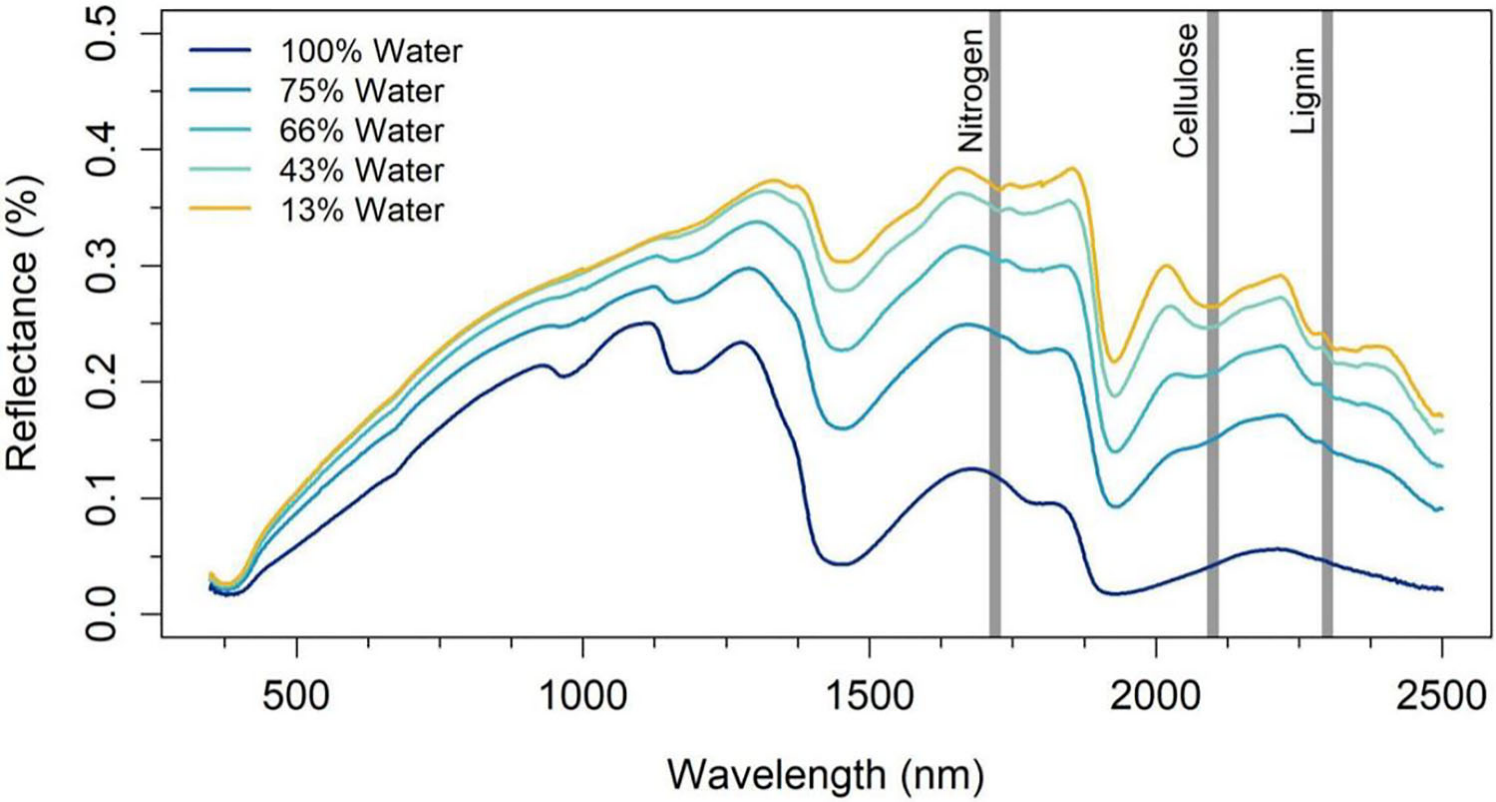
Example spectra from crop residue collected with an analytic spectral devices (ASD) spectrometer throughout a wet‐to‐dry experiment. RWC, relative gravimetricwater content.

Reflectance spectra of soils and crop residues become muted in the presence of water as less signal is provided by the darker, wetter field surface (Figure 10), explaining the reduced accuracy of the HLS‐derived NDTI index under wet conditions. We have demonstrated that the HLS‐derived SWIRA spectral angle index can be used to reduce moisture interference (Lamb et al., 2025), and collaborators have developed scene‐specific calibration adjustments using satellite‐derived surface wetness indices in conjunction with antecedent precipitation data (Rohit Nandan, oral communication, June 2025). However, these approaches are consistently outperformed by narrow‐band SWIR lignocellulose‐associated indices.

That said, narrow‐band analysis is also somewhat sensitive to interference from moisture and green vegetation (Lamb et al., 2022). From the 2015 WV3 dataset, we developed methods to adjust SINDRI‐based analysis of fCRC for moisture, using partially irrigated center pivot fields to evaluate crop residue reflectance under wet and dry conditions (Quemada et al., 2018). Future applications using the next generation of spaceborne super‐spectral and hyperspectral satellites can be used to calculate the CAI and can possibly derive sufficient information from the full spectra to correct for surface moisture, further increasing the accuracy of fCRC characterization.

#### 3.2.3 The next generation of Landsat and Sentinel satellite imagery

**FIGURE 11:**
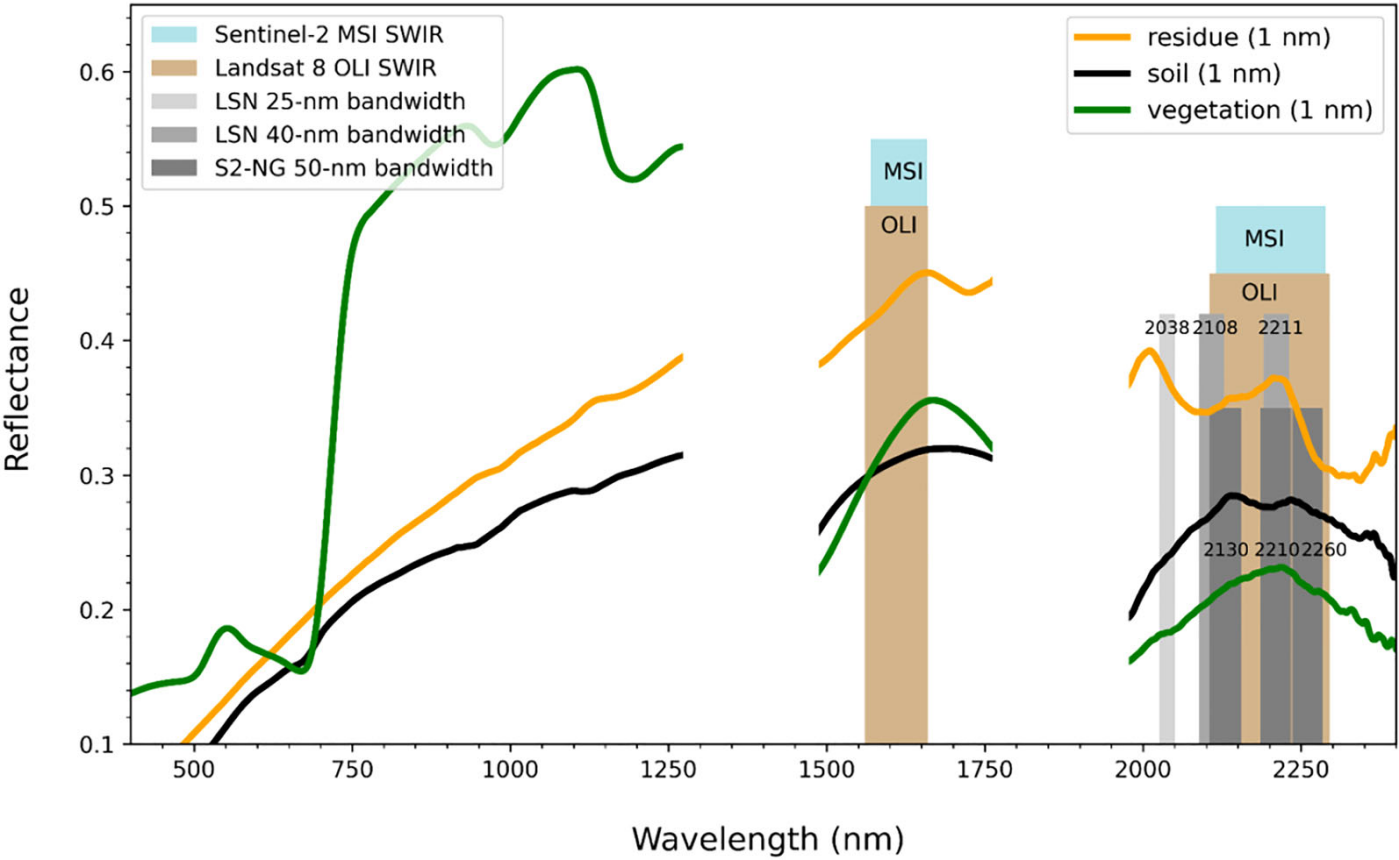
Proposed shortwave infrared (SWIR) band placement for Landsat Next (LSN) (light grey boxes) and Sentinel 2 Next Generation (S2NG) (dark gray boxes), scheduled for launch in the early 2030's. Example spectra are provided for green vegetation (green), bare soil (black), and crop residue (orange). Note the lignocellulose absorption features in the crop residue spectra from 2030 to 2300 nm. Tan and blue boxes indicate the current SWIR band specifications of the Landsat 8 Operational Land Imager (OLI) and the Sentinel‐2 Multispectral Imager (MSI), respectively. LSN and S2NG will also feature a ∼1650 nm band similar to OLI and MSI.

The next generation of multispectral satellites with global coverage will include Landsat Next (Wu et al., 2019) and Sentinel‐2 Next Generation (S2NG; Löscher et al., 2020), scheduled to launch in the early 2030s. These sensors will be “super‐spectral,” having many more narrow reflectance bands than the current generation, including three bands in the SWIR region designed to measure NPV (i.e., crop residue cover in the case of agricultural fields). The specified requirements for these three bands (i.e., the center wavelength position and bandwidth required to accurately measure NPV while maintaining sufficient signal and avoiding atmospheric absorption features) were derived in three manuscripts produced by our team, using a combination of field and laboratory ASD spectra collected at BARC (Hively et al., 2021a; Lamb et al., 2022), as well as from global spectral libraries (Dennison et al., 2023). We identified three bands suitable for measuring the CAI, centered at 2038, 2108, and 2211 nm, that were subsequently adopted by the Landsat Next mission, as well as three bands suitable for measuring the SINDRI, centered at 2130, 2210, and 2260 nm (Figure 11). Although they provide slightly reduced accuracy in measuring NPV, these latter bands were adopted by the S2NG mission because they allow a wider bandwidth to support the smaller spatial resolution of S2NG (10 m), which could not meet the recommended requirement of 25 nm bandwidth at 2038 nm to avoid atmospheric CO_2_ absorption features (Dennison et al., 2023). The S2NG band choice also provides increased continuity with the current instrument. Together, the instruments will provide global coverage at 2–3 day return frequency, enabling routine mapping of NPV and fCRC with greatly increased accuracy through direct measurement of lignocellulose absorption features.

### 3.3 Evolution of satellite remote sensing

#### 3.3.1 Hyperspectral satellites—Imaging spectrometers in space

Our science team is currently exploring applications using spaceborne imaging spectroscopy with imagery from the PRIMSA (Cogliati et al., 2021) and from the EMIT (Connelly et al., 2021; Thompson et al., 2024). These instruments record spectral reflectance in ∼10 nm increments from ∼400 to 2500 nm. They have a similar spatial resolution to HLS (30 m PRISMA, 60 m EMIT), but with a comparatively small spatial footprint and more infrequent overpass timing. An initial analysis using data collected at BARC has demonstrated improved capability for measuring cover crop performance using PRISMA, including measurement of vegetation traits that relate to decomposition dynamics (concentration of nitrogen, carbon, cellulose, starch, and lignin). In this study, laboratory spectra predicted carbon traits with *R* ^2^ of 0.86–0.98, while spaceborne imagery measured the same traits with *R* ^2^ of 0.65–0.75 (Jennewein et al., 2024). The decrease in accuracy in the field is indicative of the confounding effect of spatial variability, mixed pixels, surface moisture content, atmospheric absorption, and signal reduction.

EMIT and PRISMA imagery collection over the Delmarva Peninsula, in combination with the MDA cover crop enrollment dataset and field sampling (Jennewein et al., 2024), is providing capacity to investigate questions such as cover crop species identification, parsing weeds from cover crops, and measurement of carbon traits. The PRISMA and EMIT instruments provide capacity to accurately measure lignocellulose absorption features associated with crop residue cover while also providing spectral data that can be used to compensate for surface moisture content. They also support simulation of Landsat Next and Sentinel 2 Next Generation spectral band features to evaluate the capacity of next generation super‐spectral sensors with global coverage to map conservation outcomes.

The EMIT and PRISMA sensors are precursors to the Surface Biology and Geology (SBG) mission (Cawse‐Nicholson et al., 2021), and Copernicus Hyperspectral Imaging Mission for the Environment (CHIME; Rast et al., 2021), which will provide frequent, global coverage of high‐quality spaceborne imaging spectrometry, expected to be available by the mid‐2030s. These missions will begin a new era of Earth imaging, with increased capacity to more accurately measure environmental endpoints in the progress toward agricultural sustainability.

### 3.4 Integration with decision support tools and process‐based models

#### 3.4.1 Cover crop nitrogen dynamics

The CC‐NCALC decision support tool was designed to provide site‐specific predictions of nitrogen release during cover crop decomposition and currently requires farmer‐supplied input regarding cover crop biomass quantity and quality to inform its algorithm, which can also be remotely sensed from multi‐ and hyperspectral instruments in living and senesced cover crops. For instance, field data collected at BARC, on the Delmarva Peninsula, MO, and across the Precision Sustainable Agriculture national network (∼18 states) are being used to develop hybrid remote sensing and biophysical models to predict cover crop springtime biomass beyond the point of NDVI saturation (Jennewein et al., 2022; Prabhakara et al., 2015), by integrating accumulated incoming solar radiation (PAR) and GDD with a time series of satellite‐derived vegetation indices. Also, spaceborne hyperspectral imagery from PRISMA and EMIT is being used to quantify carbon traits from cover crop and cash crop residues (Jennewein et al., 2024), as well as algorithm development for nitrogen concentration quantification. The goal of this future work is to integrate the cover crop quantity (biomass) into the CC‐NCALC model dashboard (https://covercrop‐ncalc.org/) while developing hyperspectral algorithms to routinely quantify cover crop quality (nitrogen and carbon traits) for when routine spaceborne spectrophotometry becomes available with the launch of NASA's SBG mission and the ESA CHIME mission.

#### 3.4.2 Watershed modeling

Satellite‐based mapping of cover crop performance and of crop residue cover can inform modeling of environmental outcomes, using geospatial input to estimate the impact of conservation adoption on nutrient and sediment loss from farmlands, as well as on carbon storage and transport dynamics. Our data have been used to simulate the growth of cover crops in the SWAT model, investigating their impact on nitrogen loss under various climate scenarios (Lee et al., 2017, 2016, 2018; Yeo et al., 2013). That model was subsequently used to simulate the impact of WCC adoption in the Tuckahoe Creek sub‐watershed of the Choptank River, MD, using a combination of remote sensing of wintertime green vegetation, knowledge of MDA annual cover crop adoption in the watershed, and SWAT modeling of nitrate‐N dynamics (Hively et al., 2020). The analysis estimated a 25% reduction in bottom‐of‐field nitrate‐N leaching of N resulting from 10 years of cover crop adoption, with an estimated 38% reduction in the year with the highest adoption rates (2016: 64% of cropland area planted to cover crops).

Currently, cover crop scenarios are being incorporated into the SWAT‐Carbon model to assist in developing carbon monitoring systems for the Chesapeake Bay. The goal is to ingest regional satellite‐based mapping of cover crops and crop residue cover to inform the model, which simulates carbon transport (movement of organic matter with soil erosion or dissolved carbon through leaching) from field edge to the Chesapeake Bay estuary. Initial progress indicates that WCC adoption would noticeably increase soil carbon stocks by 0.45–0.92 MgC ha^−1^ year^−1^ on agricultural fields and contribute 2.1%–4.4% toward Maryland's 2030 greenhouse gases reduction goal if all cropland in MD used winter cover crops (Zhang et al., 2024). In addition to the carbon sequestration and water quality improvement benefits, incorporating cover crops into existing crop rotations was estimated to reduce water runoff by 5%–10% from agricultural fields in Maryland, due to increased transpiration. This finding may have implications for cover crop impacts on yield of non‐irrigated grain crops in dryland agroecosystems where water use is a limitation on crop production. Although needing confirmation in additional studies, collectively these findings highlight the importance of understanding the synergies and tradeoffs among the environmental impacts of growing winter cover crops to support sustainable agricultural development, particularly in regions that are experiencing water shortage challenges.

## 4 CONCLUSIONS

Extensive research conducted in the Mid‐Atlantic Coastal Plain landscape, in coordination with the USDA CEAP and LCB LTAR projects, has demonstrated the utility of satellite remote sensing for monitoring the performance of agricultural conservation practices in the working farm landscape. No‐cost multispectral HLS imagery is used to measure cover crop biomass, nitrogen content, and fractional ground cover, as well as estimate green‐up and termination dates, for every field enrolled in the MACS cost‐share program (>25,000 per year). Results are used by MDA to streamline program management, including adaptive management of variable incentive rates to promote effective outcomes. Finer resolution PlanetScope imagery has demonstrated utility for measuring cover crop performance on small fields and research trials. Meanwhile, work is underway to develop applications of spaceborne imaging spectroscopy to identify cover crop species, weeds, and cover crop carbon traits (i.e., forage quality). High‐spectral resolution imagery has been shown to accurately mappin fractional crop residue cover, and research results have guided design specifications for the Landsat Next and Sentinel 2 Next Generation satellites, which will provide wall‐to‐wall capacity for measuring NPV by the 2030s.

These remote sensing outputs are used in decision support tools and modeling efforts to estimate changes in nutrient and sediment loss and carbon cycling resulting from implementation of conservation practices in the working farm landscape. When developed in collaboration with conservation program managers, remote sensing data products can support adaptive management of conservation incentive programs to increase environmental outcomes. The USDA CEAP and LCB LTAR projects have helped to develop these outcomes by providing tools for cropland conservation management in the working farm landscape.

## AUTHOR CONTRIBUTIONS

**W. Dean Hively**: Conceptualization; formal analysis; funding acquisition; investigation; methodology; project administration; resources; supervision; visualization; writing—original draft; writing—review and editing. **Feng Gao**: Conceptualization; investigation; methodology; validation; visualization. **Gregory W. McCarty**: Conceptualization; funding acquisition; project administration; resources. **Craig S. T. Daughtry**: Conceptualization; investigation; methodology. **Xuesong Zhang**: Conceptualization; investigation; methodology; writing—original draft. **Jyoti Jennewein**: Conceptualization; data curation; formal analysis; investigation; methodology; validation; visualization; writing—review and editing. **Alison Thieme**: Data curation; formal analysis; methodology; validation; visualization; writing—original draft. **Brian T. Lamb**: Data curation; formal analysis; investigation; methodology; validation; visualization; writing—review and editing. **Jason Keppler**: Conceptualization; methodology; resources. **Cathleen J. Hapeman**: Funding acquisition; project administration; writing—original draft. **Michael Cosh**: Funding acquisition; project administration; writing—review and editing. **Steven B. Mirsky**: Project administration; writing—review and editing.

## CONFLICT OF INTEREST STATEMENT

The authors declare no conflicts of interest.
